# Phylogenetic Analysis of Symbiotic Bacteria Associated with Two *Vigna* Species under Different Agro-Ecological Conditions in Venezuela

**DOI:** 10.1264/jsme2.ME19120

**Published:** 2020-01-11

**Authors:** María Daniela Artigas Ramírez, Mingrelia España, Sylwia Lewandowska, Kun Yuan, Shin Okazaki, Naoko Ohkama-Ohtsu, Tadashi Yokoyama

**Affiliations:** 1 Institute of Global Innovation Research, Tokyo University of Agriculture and Technology (TUAT), Saiwai-cho 3–5–8, Fuchu, Tokyo—Japan; 2 Institute for Advanced Studies (IDEA), Miranda—Venezuela; 3 Department of Genetics, Plant Breeding and Seed Production, Wrocław University of Environmental and Life Sciences, 50–363 Wrocław, 24A, Grunwaldzki—Poland; 4 United Graduated School of Agriculture, Tokyo University of Agriculture and Technology (TUAT), Saiwai-cho 3–5–8, Fuchu, Tokyo—Japan; 5 Institute of Agriculture, Tokyo University of Agriculture and Technology (TUAT), Saiwai-cho 3–5–8, Fuchu, Tokyo—Japan

**Keywords:** *Ensifer*, *V. unguiculata*, *Burkholderia*, *Bradyrhizobium*, Venezuela

## Abstract

*Vigna* is a genus of legumes cultivated in specific areas of tropical countries. Species in this genus are important crops worldwide. *Vigna* species are of great agronomic interest in Venezuela because *Vigna* beans are an excellent alternative to other legumes. However, this type of crop has some cultivation issues due to sensitivity to acidic soils, high temperatures, and salinity stress, which are common in Venezuela. *Vigna* species establish symbioses mainly with *Bradyrhizobium* and *Ensifer*, and *Vigna*-rhizobia interactions have been examined in Asia, Africa, and America. However, the identities of the rhizobia associated with *V. radiata* and *V. unguiculata* in Venezuela remain unknown. In the present study, we isolated Venezuelan symbiotic rhizobia associated with *Vigna* species from soils with contrasting agroecosystems or from fields in Venezuela. Several types of soils were used for bacterial isolation and nodules were sampled from environments characterized by abiotic stressors, such as high temperatures, high concentrations of NaCl, and acidic or alkaline pH. Venezuelan *Vigna*-rhizobia were mainly fast-growing. Sequencing of several housekeeping genes showed that in contrast to other continents, Venezuelan *Vigna* species were nodulated by rhizobia genus including *Burkholderia*, containing bacteria from several new phylogenetic lineages within the genus *Bradyrhizobium*. Some *Rhizobium* and *Bradyrhizobium* isolates were tolerant of high salinity and Al toxicity. The stress tolerance of strains was dependent on the type of rhizobia, soil origin, and cultivation history. An isolate classified as *R. phaseoli* showed the highest plant biomass, nitrogen fixation, and excellent abiotic stress response, suggesting a novel promising inoculant for *Vigna* cultivation in Venezuela.

*Vigna* is a genus in the legume family (Fabaceae), and some of the oldest crops known to man belong to the genus *Vigna*. *Vigna* originated in Africa and were introduced to the Americas from Eastern Africa in 1500 AD (Doyle, 1994). They are essential nutritional legumes, and a valuable component of the traditional cropping systems found in semi-arid tropical regions, such as Asia, Africa, and Central and South America (Singh *et al.*, 1997; Zhang *et al.*, 2008; Sarr *et al.*, 2011). There are approximately 11 million hectares under *Vigna* cultivation worldwide. Asian countries are responsible for the majority of global *Vigna* production, accounting for 90% of 5.8 million tons of *Vigna* beans annually (Zhang *et al.*, 2008; Risal *et al.*, 2012).

*Vigna* is a widespread genus and is part of the third-largest family of flowering plants (Fabaceae) (Allen and Allen, 1981; Doyle, 1994; Lambrides and Godwin, 2007). Legume members of *Vigna* consist of approx. 20,000 species (Doyle, 1994). This genus contains several species that are important in the agricultural world, such as mung beans (*V. radiata*), cowpea (*V. unguiculata*), adzuki beans (*V. angularis*), urad beans (*V. mungo*), rice beans (*V. umbellata*), moth beans (*V. aconitifolia*), and bambara groundnut (*V. subterranea*) (Lambrides and Godwin, 2007). In Venezuela, there is great diversity in *Vigna* cultivars, including *V. unguiculata* and *V. radiata*, which are important components of the Venezuelan diet (Vásquez, 2014). These species are commonly called “Frijol bayo” or “Frijol chino” in local colloquial language (Vásquez, 2014). *Vigna* legumes are a good alternative to other legumes present in the Venezuelan diet. *V. unguiculata* is of great agronomic interest due to its resistance to soil acidity, drought, and high temperatures (Timko *et al.*, 2007; Li *et al.*, 2009). However, *V. radiata* may develop major issues during cultivation in Venezuela due to its low tolerance to high temperatures, drought, and some edaphological conditions, which reduce production, in addition to disease and pest problems (Lambrides and Godwin, 2007; Vásquez, 2014).

*Vigna*-nodulating rhizobia are genetically diverse (Risal *et al.*, 2012). They were previously characterized as slow-growing bradyrhizobia (Allen and Allen, 1981). *Vigna* has‍ ‍been characterized for establishing symbioses in most countries, mainly with bradyrhizobia. These bradyrhizobia-legume interactions have been examined in Asia, Africa, and the Americas (Lambrides and Godwin, 2007; Risal *et al.*, 2012). Recent studies reported that some *Vigna* species also successfully establish N_2_-fixing symbiosis with *Ensifer* and *Rhizobium* species (Lu *et al.*, 2009; Ren *et al.*, 2011; Risal *et al.*, 2012; Andrews and Andrews, 2017). However, some rhizobia, such as *Mesorhizobium*, *Burkholderia*, and *Microvirga*, display host specificity with *Vigna* (Lu *et al.*, 2009; Radl *et al.*, 2014; Andrews and Andrews, 2017).

In Venezuela, the rhizobia genetically associated with *Sesbania* sp. have been characterized as *Leucaena* sp., *Medicago sativa*, and *Phaseolus vulgaris* (Vinuesa *et al.*, 2005; Marquina *et al.*, 2011). In 2018, Venezuelan soybean rhizobia were characterized and belonged to *Rhizobium* with a predominance of the genus *Burkholderia* (Artigas *et al.*, 2019). However, *Vigna*-rhizobia symbiosis has not yet been examined under Venezuelan environmental conditions. The present study was conducted to elucidate the genetic diversity and geographical distribution of *Vigna*-rhizobia under different Venezuelan agro-conditions as well as the symbiotic functioning of Venezuelan isolates and *Vigna* species. The results obtained may contribute to the search for the best bacteria for crop inoculation and increased crop production under Venezuelan conditions.

## Materials and Methods

### Collection sites and soil samples

**Fig. 1.**
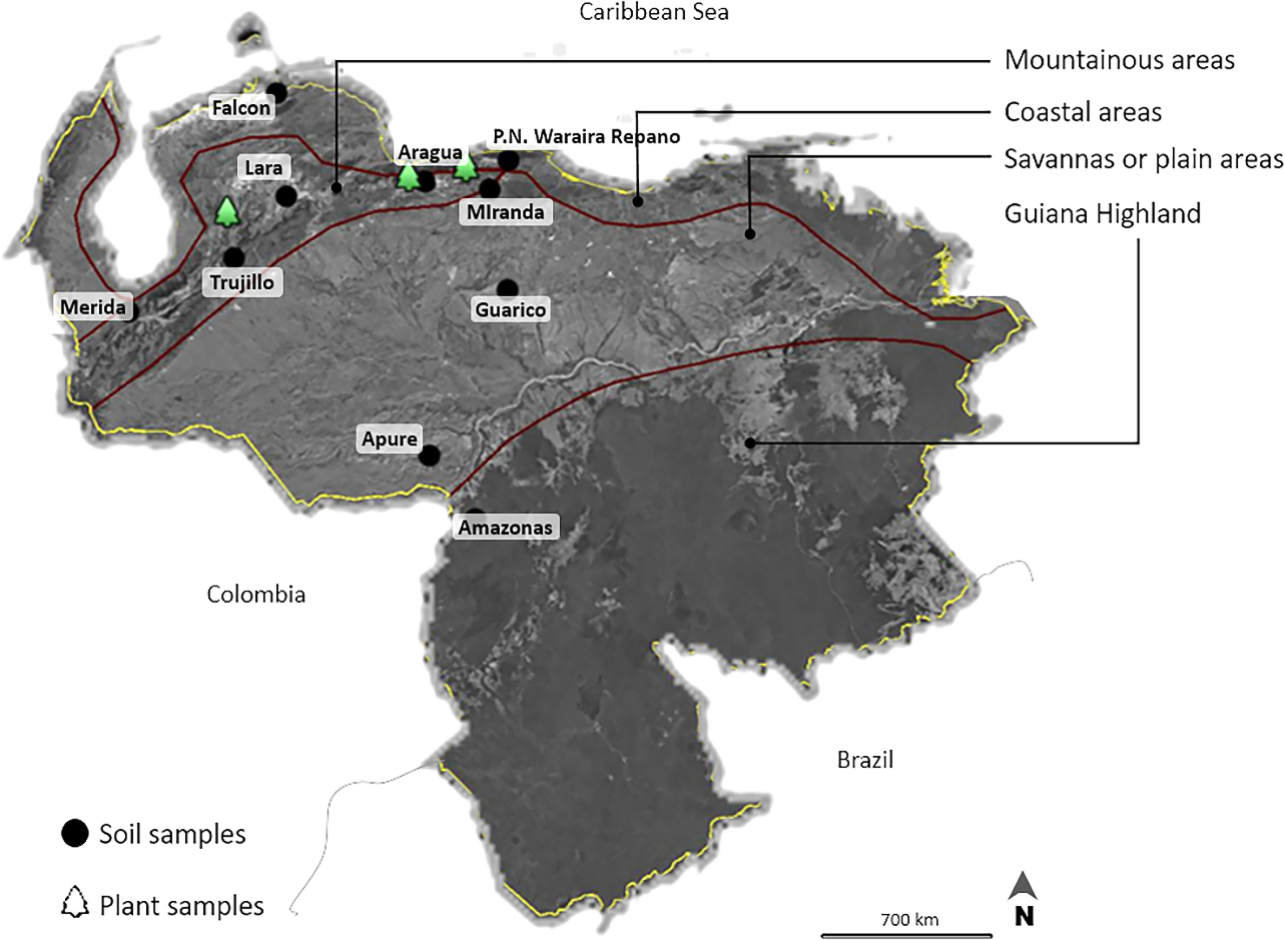
Map of Venezuela showing different agro-ecological regions for collection sites, and geographical locations of soil and plant samples used for rhizobial isolation (This map was made by Google earth Pro software).

Soil samples were collected from 10 Venezuelan regions and root nodules were picked up from 3 areas in which *Vigna* had previously been cultivated (Fig. 1 and Table 1). These areas were located in diverse agroecological regions with contrasting climates, topographies, and soils (Table 1). Soil samples from each area were a composite of 2 sub-samples prepared by mixing soils obtained from depths of 0–20 cm. No bacterial inoculations had previously been performed in these areas. Therefore, these strains were considered to be indigenous to Venezuela. Furthermore, soil samples were collected from different types of soils, including Alfisol, Oxisol, Inceptisol, Aridisol, Ultisol, and Vertisol (Table 1).

### Isolation of rhizobia from Venezuelan soils using *Vigna* species as trap hosts

Two types of *Vigna* species were used in the present study: the cowpea *V. unguiculata* cultivar ‘Tuy’ (Venezuelan variety) and the mung bean *V. radiata* cultivar ‘Ryokutou’ (Japanese variety). *V. unguiculata* and *V. radiata* were used in inoculation tests (pot tests) with soils from different agroecological conditions. Seeds were surface-sterilized and inoculated with 5-fold dilutions of soil suspensions (Risal *et al.*, 2012). After the seeds were sown, plants were grown in a growth room chamber under sterile conditions. A sterilized nitrogen-free nutrient solution (Somasegaran and Hoben, 1994) was added to the glass jars (300 mL). A moisture level of 60% of the water field capacity was established and maintained throughout the growth period. Plants were grown for 4‍ ‍weeks in the growth chamber under a 16-h light (5,000~7,000 LUX) and 8-h dark photoperiod at 28°C. After four weeks, the root nodules were collected and washed with sterile distilled water. *V. unguiculata* root nodules were also harvested from field conditions (as dried nodules, Table 1).

The root nodules were sterilized as described previously (Artigas *et al.*, 2019). After sterilization, the root nodules were washed with sterile distilled water 4 times. Surface-sterilized root nodules were crushed in 500 μL glycerol solution (15% [v/v]) to obtain a bacterial suspension. An aliquot (10 μL) of the suspension was streaked (Vincent, 1970) onto 1.5% (w/v) Yeast Mannitol agar (YMA) medium (Somasegaran and Hoben, 1994). The agar plates were then incubated at 28°C for one week. The remaining suspension was frozen at –80°C for further isolations, if necessary. Single colonies were restreaked onto fresh agar plates to obtain pure colonies. Phenotypically, the strains were characterized in terms of their growth rate, texture, and color on YMA plates. These isolates were re-inoculated onto the host plant to verify their nodulation abilities.

### Stress tolerance screening

Isolates were evaluated for growth under different abiotic stress conditions: high temperature, alkaline pH, acidic pH, high salinity, and a high concentration of aluminum (Al) at different pHs. The temperature tolerance of isolates was tested by recording their ability to grow under the following temperatures: 25, 28 (control), 35, and 40°C on YMA plates. Regarding pH tolerance, the abilities of isolates to grow at different pHs was tested under the following pH conditions: 4.5, 5, 6.8 (control), 8, and 10 (Somasegaran and Hoben, 1994). These tests were performed on YMA plates with pH adjusted by 0.5 M HCl or 0.5 M NaOH. The salinity and Al tolerance of isolates were assessed as described previously (Artigas *et al.*, 2019). The isolates were initially grown in YM broth for five d at 28°C, and 5 μL of cell suspensions at 10^9^‍ ‍cells‍ ‍mL^–1^ were ten transferred to YMA plates and YM broth under stress conditions at 28°C for 4–10 d. The growth of strains on YMA was estimated relative to the control treatment, as described previously (Artigas *et al.*, 2019).

### Plant tests for symbiosis analysis

A total of 120 isolates were selected as representatives of diverse groups from abiotic stress tolerance assays, which included sensitive and tolerant strains. Pre-selected isolates were grown in YM broth for five d at 28°C to obtain 10^7^‍ ‍cells‍ ‍mL^–1^, as described previously (Vincent, 1970). Prior to the inoculation, the seeds of *V. unguiculata* ‘Tuy’ were surface-sterilized with 70% ethanol for 30‍ ‍s, 3% (v/v) of sodium hypochlorite for 2 min, and then washed 4 times with sterile distilled water. Seeds were then inoculated by soaking in these rhizobial cells at 10^7^‍ ‍cells‍ ‍mL^–1^ and sown on 200 g of vermiculite (Vermitech) in a plant box (7.6×7.6×10.2 cm). A sterilized nitrogen-free nutrient solution (Sylvester–Bradley *et al.*, 1983) was added to vermiculate to reach a moisture level that was 60% of the field capacity, and this level was maintained throughout the growth period. Plants were cultivated for 30‍ ‍d in the growth chamber (FLI 2000—EYELA; Tokyo Rikakikai Corporation, Tokyo, Japan) under a 16-h light/8-h dark photoperiod at 28°C. Three replicates per strain were performed for all treatments, and one replicate contained one plant per plant box. Non-inoculated plants served as control treatments (Vincent, 1970). To obtain an accurate mass, these root nodules, shoots, and roots were dried at 80°C for 48 h before weighing.

### Nitrogenase activity

Intact plants with root nodules were collected after 30‍ ‍d of culture for nitrogen fixation assessments using an acetylene reduction assay (ARA). ARA was performed using a Shimadzu gas chromatograph GC-2014 gas chromatograph (Shimadzu Corporation, Kyoto, Japan) equipped with a Porapak N column (Agilent Technologies, Santa Clara, USA). Whole root systems of *Vigna* plants were placed into an incubation bottle (300 mL) with a sealed cap, and 10% (v/v) of the air was replaced with acetylene gas. Samples were incubated at 28°C for 30–40 min. After the incubation, 1 mL of the gas sample was injected into the gas chromatograph, and this was followed by the assessment of root nodule numbers. The masses of shoots, roots, and root nodules were measured after they were dried at 80°C for 48 h.

### Statistical analysis

Statistical analyses were performed with Tukey’s and Dunnett’s tests using Statistica software version 12.0 (StatSoft, Tulsa, USA). A value of *P*≤0.05 was considered to be significant.

### Isolation of genomic DNA

Forty-six isolates were selected based on their symbiotic performance and tolerance to abiotic stress. DNA was extracted from isolates grown in YM broth medium at 28°C for four d. Prior to DNA isolation, cells were collected and washed twice with equal volumes of TE buffer. Total genomic DNA was extracted as described previously (Artigas *et al.*, 2019).

### DNA amplification and sequencing

PCR amplification and sequencing of 16S rRNA, *atpD*, *nod*, and *nif* gene regions were performed as described previously (Risal *et al.*, 2012). The primer sets used for the PCR of 16S rRNA and *nodD* genes were described previously (Risal *et al.*, 2012). The *atpD* (ATP synthase) primer set used was described by Gaunt *et al.* (2001). The *nifH* primer set for the *nif* gene was described previously by Laguerre *et al.* (2001). Amplifications were performed as described by Artigas *et al.* (2019). Thermal cycling conditions were as follows: denaturation at 95°C for 4 min, 35 cycles of denaturation at 94°C for 1 min, annealing at 60 or 55°C for 45 s, and extension at 72°C for 2 min, followed by a final extension at 72°C for 5 min. Amplifications were performed using a thermal cycler (GeneAmp PCR system 9700; Applied Biosystems, Waltham, USA). PCR products were checked using agarose gels, and DNA was purified using a Fast Gene Gel/PCR extraction kit (Nippon Genetics, Tokyo, Japan) for all genes. PCR products were sequenced using an ABI Prism 3500 Genetic Analyzer (Applied Biosystems), according to the manufacturer’s protocol. The sequences obtained were compared with the corresponding genes deposited in the GenBank database (https://www.ncbi.nlm.nih.gov/genbank/) using the online software BLAST algorithm-based sequence alignment. Phylogenetic trees were constructed using the software Genetix version 11 and MEGA version 6.0 (Tamura *et al.*, 2013).

### Accession numbers

The gene sequences obtained in the present study were deposited in the DNA Databank of Japan (DDBJ) under accession numbers LC460871 to LC460916 (16S rRNA), LC460917 to LC460962 (*atpD* gene), LC461082 to LC461127 (*nifH* gene), and LC461128 to LC461173 (*nodD* gene).

## Results

### Characterization of *Vigna* rhizobia isolated from different Venezuelan soils

The characteristics of soils used for rhizobia-isolation included Al concentrations shown in Table 1. In the present study, acidic soils were taken from the Venezuelan Andes (Trujillo and Mérida sites). Trujillo was the sampling site with the highest Al concentration and lowest pH in this study. Venezuelan savannas, located in Guárico and Apure, also had low pH. Additionally, the rainforest of the Amazonas State possessed Oxisol with an acidic pH. Guárico and Falcón showed the highest temperatures of approximately ≥35°C (Table 1).

The total number of root nodules was 623, with 523 being harvested from pot experiments with soils and 100 collected from field-grown *Vigna* species (Valley and Andes sites, Table 1). Among the *Vigna* plants used as trap hosts, 475 root nodules were obtained from the Venezuelan ‘Tuy’ cultivar of *V. unguiculata* (cowpea) and 148 were harvested from the Japanese ‘Ryokutou’ cultivar *V. radiata* (mung bean), as shown in Table 1. The Aragua—Valley, located in north-central Venezuela, was classified as an area that cultivated crops with or without the application of inorganic fertilizers (Table 1). This sampling site produced the most abundant root nodules. We sampled 54 root nodules from the Amazonas site, located in the rainforest ecosystem. This site is located in the Guiana Highlands, which traditionally produces crops such as cucumber, tomato, and coriander. It is important to note that *V. radiata* showed no nodulation at the Apure, DC, and Trujillo sites. In Aragua and Trujillo, 9 strains of *V. unguiculata* were successfully isolated from dried nodules (field collection). A total of 287 strains were isolated from both *Vigna* species (Table 1).

### Physiological characterization of *Vigna* rhizobia under abiotic stress conditions

The physiological properties of the 287 isolates were evaluated under abiotic stress. Furthermore, a representative group of isolates (120 strains) was selected according to their tolerance of and sensitivity to different abiotic stresses (Table 2 and S1). The growth rates of these isolates were classified into two groups: fast growers represented 41% and intermediate growers 59% (Table S1). Isolates were distinguished phenotypically by morphological characteristics, such as color and texture. Total isolates were classified into four types according to color: being predominant white-transparent color (WT; *n*=102). Accordingly, the strains were dominant by a creamy texture in 104 isolates (Table S1).

Strains showed different abilities to grow under high-temperature conditions. Eighty-seven percent of isolates were able to grow at 40°C, showing high-temperature tolerance (Table S1). In the case of salinity tolerance, all isolates grew similar to or better than the control (0%) at 1% NaCl. Nine isolates (3% of the total) did not grow at the same rate with 2% NaCl, and also did not grow under 3 and 4% NaCl. Ninety-five percent of isolates tolerated high concentrations of NaCl (4%). Some isolates grew under 3 and 4% NaCl conditions and grew similar to or better than the control (0% NaCl). At 4% NaCl, the isolates from Apure (10 isolates), Guárico (12 isolates), and Falcón (9 isolates) grew similar to the control. Two isolates from Amazonas (AmR5) and Aragua (valley with fertilizer) (AFV3) did not grow at 4% NaCl (Table S1).

Most of the strains were tolerant to alkaline conditions, but sensitive to acidic conditions. Eighteen percent of isolates did not grow under acidic conditions, whereas all isolates successfully grew under alkaline conditions; however, 2% showed weaker growth at pH 10 than controls at pH 6.8 (Table S1). In contrast, eight isolates did not survive under acidic conditions (pH 4.5), particularly those from alkaline soils, such as Falcón and Aragua. For example, two isolates from Merida and Trujillo showed weaker growth than the control (pH 6.8). Similarly, the growth of isolates was severely inhibited at high Al concentrations, but was inhibited more under acidic pH. Al toxicity is lower under neutral pH conditions because nearly 98% of isolates survive and grow under these conditions. Accordingly, isolates from soils with Al or acidic soils showed more tolerance to Al toxicity, such as Trujillo. Nine percent of isolates grew under a combination of 2‍ ‍mM of Al and pH 4.5 (Table S1).

These isolates (120 strains) were inoculated into *V. unguiculata* seeds. However, only 46 isolates exhibited nodulation activity on *V. unguiculata*. The physiological characteristics of selected isolates are summarized in supplementary Table S1 (highlighted with positive nodulation activity). These nodulating isolates grew under high temperatures, high concentrations of NaCl, and an alkaline pH (Table S1). Only two isolates did not survive in acidic soils: one strain from Aridisol (Falcon, FV3) and the other from Ultisol (Trujillo, TrV2B). Nine fast-grower isolates were Al-tolerant at 2‍ ‍mM combined with 4.5 pH. These isolates were subsequently analyzed according to their genetic characteristics and symbiotic performance.

### Distribution of rhizobia and phylogeny based on 16S rRNA and *atpD* genes

**Fig. 2.**
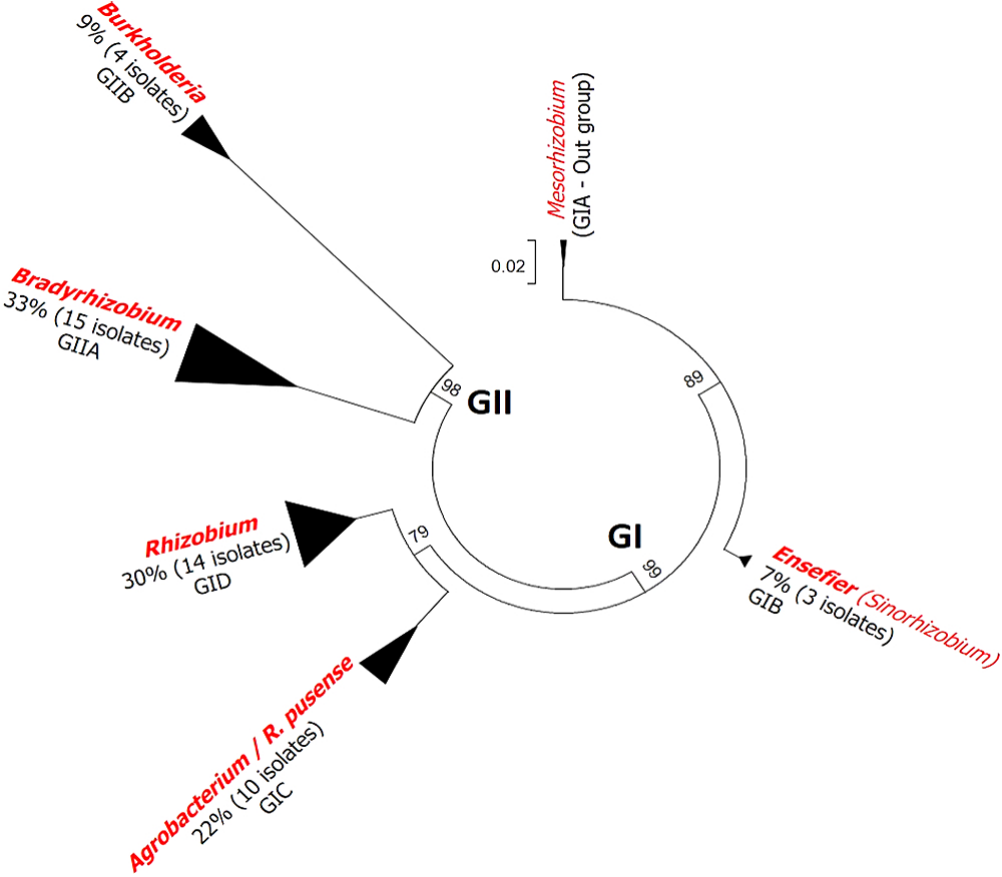
General analysis based on 16S rRNA sequences of Venezuela isolates. The phylogenetic tree included *Vigna*-rhizobia (46 isolates) and references of α-rhizobia and β-rhizobia (38 reference strains). The tree is based on differences in 1,390-bp DNA fragments. The scale bar represents substitutions per nucleotide position and each genus included the percentage of total isolates. Numbers at the nodes indicate the level of bootstrap support (%), based on a neighbor-joining analysis of 1,000 re-sampled datasets.

Forty-six isolates were analyzed using the 16S rRNA gene, their general taxonomic position, and their distribution, as shown in Table 2. The phylogenetic analysis clearly showed that Venezuelan isolates clustered into two major groups of bacteria: *α-Proteobacteria* and *β-Proteobacteria* (Table 2). These isolates were classified as follows: group I (GI) contained 58% of all isolates (42 α-rhizobia), which were widely distributed in Venezuela, and group II (GII) included α-rhizobia and β-rhizobia. Thirty-three percent of all isolates were classified as *Bradyrhizobium*, while the genus *Burkholderia* represented 9% (Fig. 2). Their main distribution in Venezuela was as follows: strains from Aragua (Inceptisol soil) had a high level of *Vigna*-rhizobia (Table 2). Although, there is a specificity in Falcón (Aridisol) where the isolates were classified as *Ensifer. Agrobacterium*/*R. pusense* included ten isolates from different ecosystems, such as template (Mérida) and rainforest (Amazonas). All isolates from Trujillo belonged to the genus* Bradyrhizobium*. β-rhizobia isolates were mainly from Vertisol (3 isolates) and one was from Aridisol (Table 2).

**Fig. 3.**
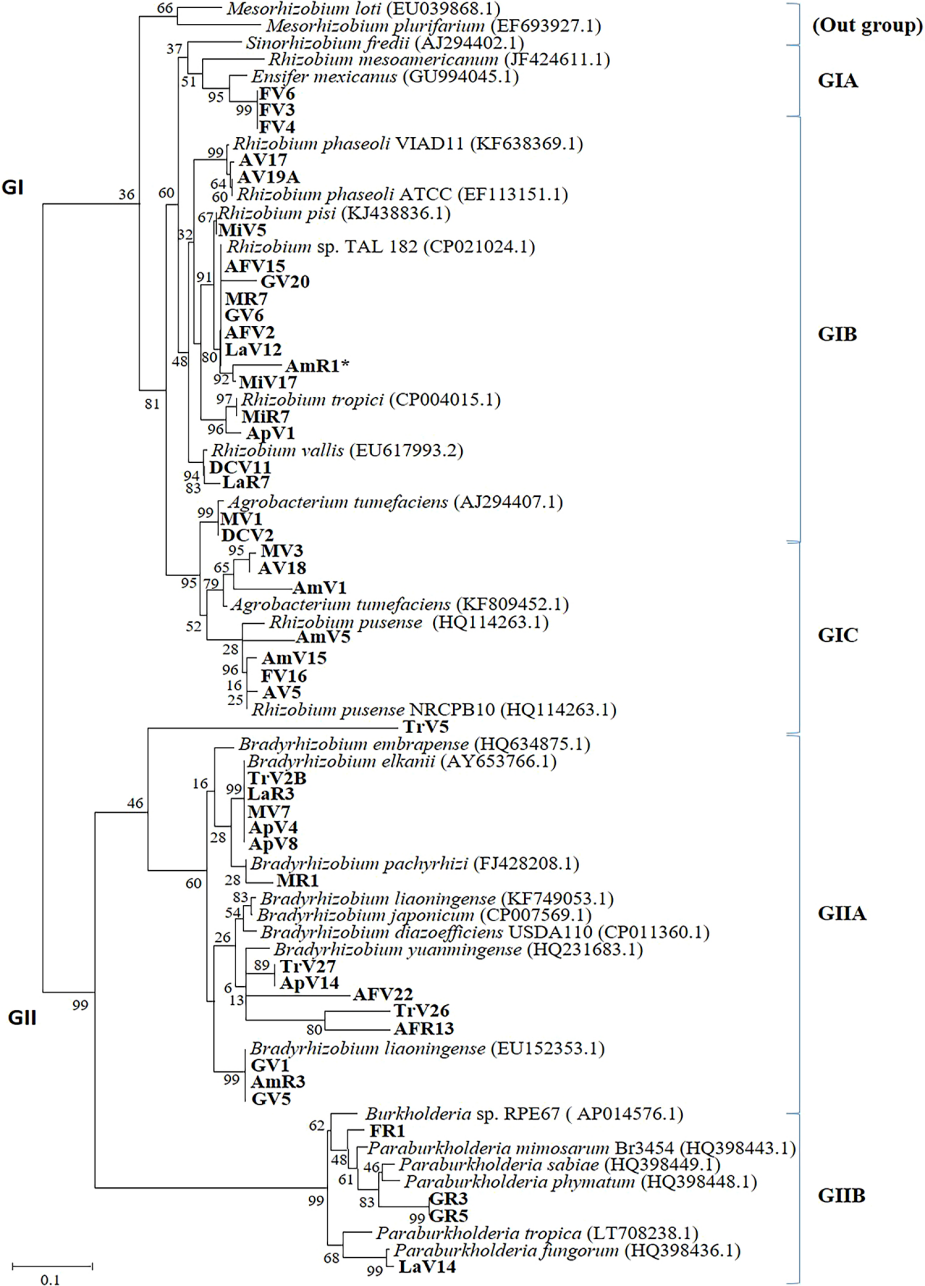
Phylogenetic analysis of *atpD* as the housekeeping gene for Venezuelan isolates. The phylogenetic tree included *Vigna*-rhizobia (46 isolates) and references of α-rhizobia and β-rhizobia (29 reference strains). The tree is based on differences in 500-bp DNA fragments. The scale bar represents substitutions per nucleotide position and each genus included the percentage of the total isolates. The isolate name includes the sampling site and associated seed variety.

To confirm the results obtained, one housekeeping gene (*atpD*) was phylogenetically analyzed (Fig. 3). The results obtained were generally consistent with 16S rRNA gene results. Accordingly, Venezuelan isolates closely related to α-rhizobia were mainly classified into the genera *Rhizobium* and *Bradyrhizobium* (Fig. 3 and S1). GI contained the out-group *Mesorhizobium*, which did not have a close relationship with any isolates from this study. Three isolates were classified as *E. mexicanus* (GIA). GIB grouped different *Rhizobium* species and 14 Venezuelan isolates. The relationship between isolates and *Rhizobium* species was as follows: *R. etli* (4 isolates), *R. phaseoli* (4 isolates), *R. vallis* (1 isolate), *R. tropici* (2 isolates), and *R. pisi* (1 isolate), and one isolate included as *Rhizobium* sp. (Fig. S1). These results suggested that *Agrobacterium* and *R. pusense* were closely related, and these genera were grouped into the same clade (GIC) with Venezuelan isolates.

In the second group of *Vigna*-rhizobia isolates (GII), 15 isolates were classified as the genus *Bradyrhizobium* (GIIA). Four isolates had a close relationship with *B. embrapense*, while *B. elkanii* and *B. pachyrhizi* were also closely related to 3 isolates. Isolates within this sub-clade were closely related to *B. rifense* (1 isolate), *B. yuanmingense* (2 isolates), *B. japonicum* (1 isolate), and *B. liaoningense* (3 isolates). It is important to note that one isolate, TrV5, had no related reference strain, which suggests that this is a novel genospecies or symbiovar of *Bradyrhizobium*. In the case of β-rhizobia, isolates were closely related to *Burkholderia* species (GIIB). These isolates were related to *Paraburkholderia symbiont* and *P. tuberum* (Fig. S1). The last isolate had no related reference strain. Based on *atpD*, these isolates were all confirmed to be *Paraburkholderia*.

Moreover, small incongruences were found between 16S rRNA and *atpD* genes. For example, AFV2, AmR1, and MiV17 were classified into a small sub-group separate from *R. phaseoli* or *R. etli* (Fig. S1), while based on *atpD* sequences, isolates were reclassified as *Rhizobium* sp. TAC182^T^ (Fig. 3). LaR7 (isolated from Lara) was identified as *Agrobacterium/R. pusense*. LaR7 was reclassified as *R. vallis*. In the *Bradyrhizobium* group (GIIA), three isolates (AFR13, TrV27, and TrV26) were re-grouped as *B. yuanmingense* using *atpD* sequences.

### Phylogenetic analysis based on *nodD* and *nifH* gene sequences

**Fig. 4.**
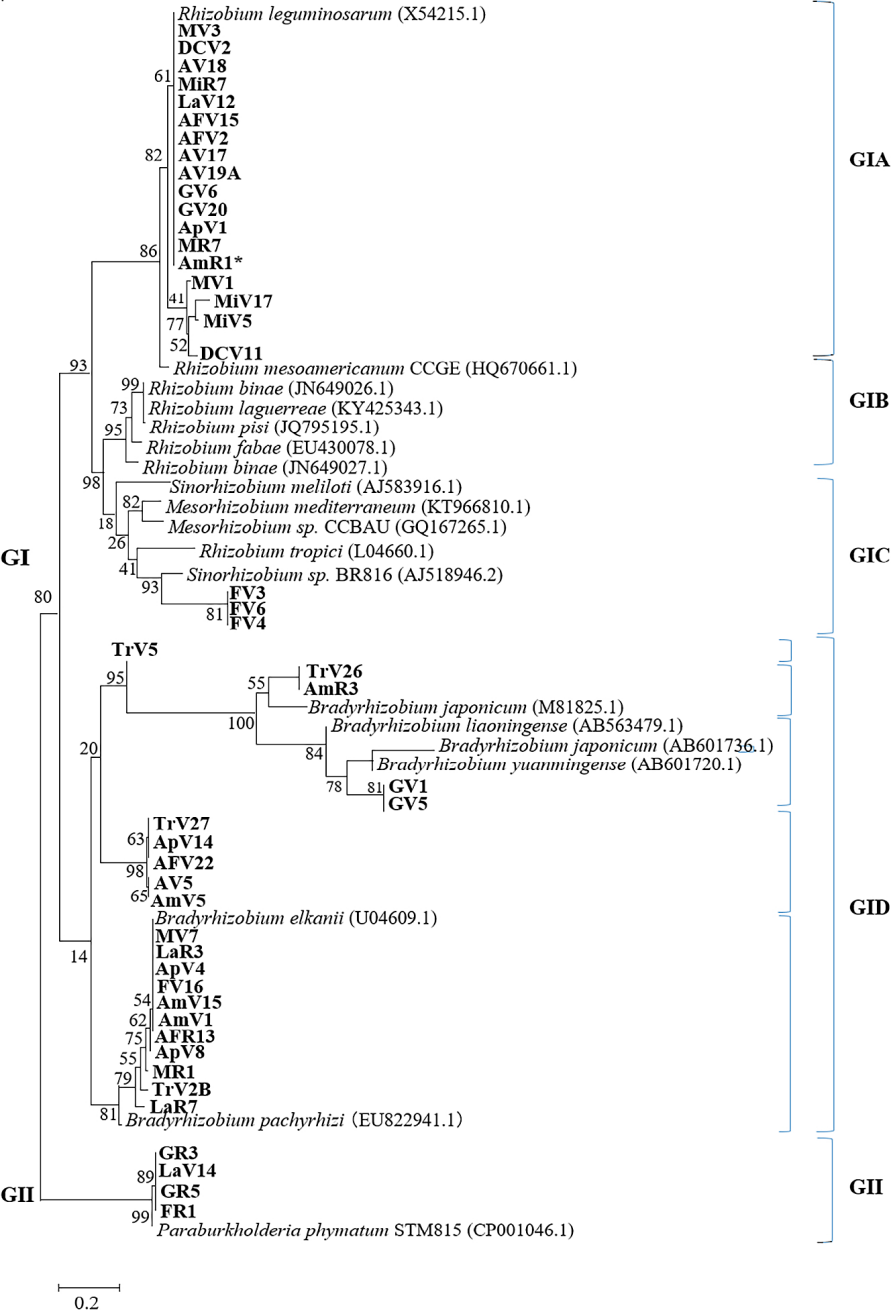
Phylogenetic analysis based on *nodD* sequences of Venezuelan isolates. Differences are based on a 690-bp DNA fragment of the *nodD* gene obtained from *Vigna* rhizobia. The numbers at the branch nodes indicate bootstrap values (%), based on a neighbor-joining analysis of 1,000 re-sampled datasets. The scale bar indicates substitutions per site.

A summary of the phylogenetic groups of *Vigna* isolates is shown in Table 3. Forty-two isolates were grouped in GI (α-rhizobia) and the remaining isolates were β-rhizobia GII (Fig. 4). The first group was divided into the following five subgroups. GIA has a close relationship with the *nodD* gene that originated from *R. leguminosarum*. This group included all isolates classified previously as *Agrobacterium*/*R. pusense*. GIB was an outgroup. *Ensifer* and three isolates were grouped into GIC; these isolates were previously classified as *E. mexicanus*. Bradyrhizobia were classified into GID, which included all bradyrhizobia isolates, and subdivided into five subgroups according to the references as follows: *B. japonicum* (2 isolates), *B. yuanmingense* (2 isolates), *and B. elkanii* (11 isolates). The remaining bradyrhizobia isolates included seven isolates. These isolates were previously classified as *Ensifer* (one isolate) and *R. pusense* (six isolates) (Fig. 4 and Table 3). The *nodD* sequence analysis showed that β-rhizobia isolates were grouped as *P. phymatum* (GII). However, based on other sequences, these isolates were classified into different *Paraburkholderia* species.

**Fig. 5.**
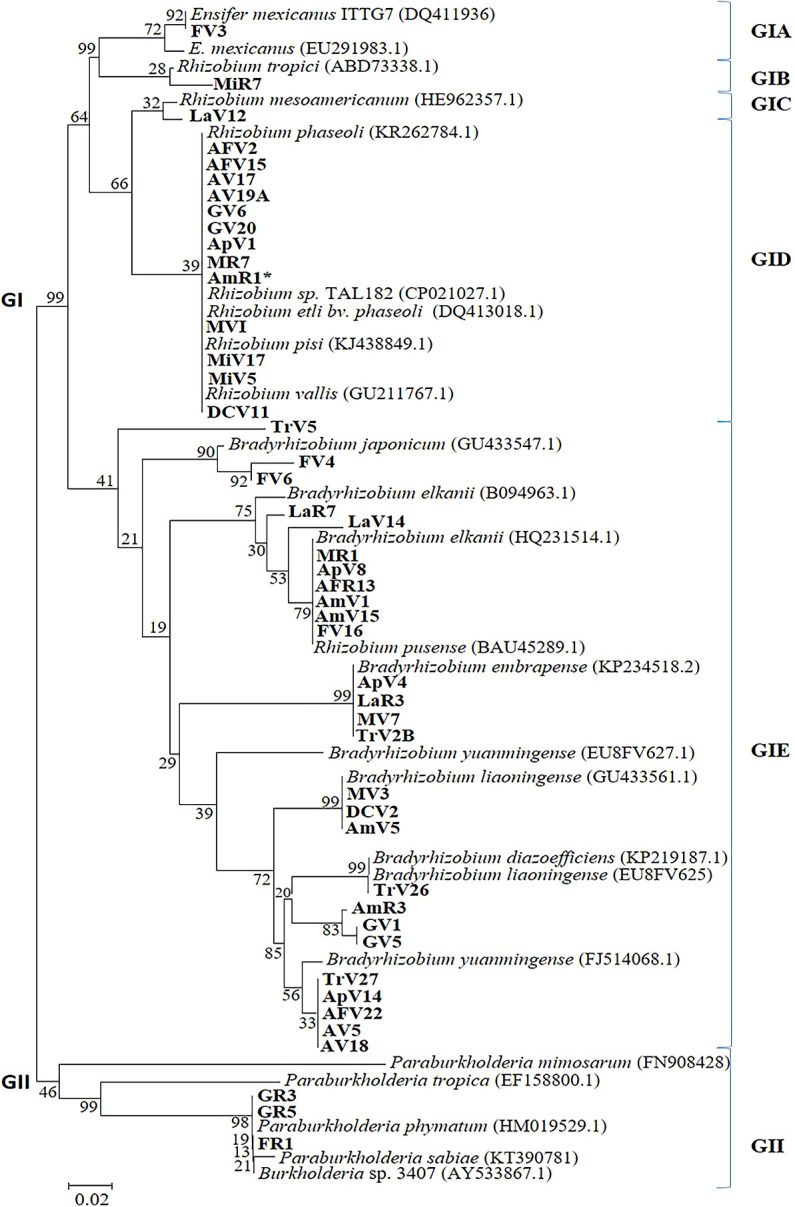
Phylogenetic tree based on *nifH* sequences of *Vigna* isolates. The tree is based on differences in 750-bp DNA fragments. The numbers at the branch nodes indicate bootstrap values (%) based on a neighbor-joining analysis of 1,000 re-sampled datasets. The scale bar indicates 0.02 changes per site.

The *nifH* phylogenetic analysis showed similar results to the *nodD* sequences (Fig. 5 and Table 3). All isolates were grouped into GI (α-rhizobia) and GII (β-rhizobia). GI was divided into five subgroups: GIA included one isolate (FV3) and *E. mexicanus*. *R. tropici* and MiR7 were classified as GIB. The LaV12 isolate was regrouped with *R. mesoamericanun* into GIC. GID is one of the largest groups that contained 13 Venezuelan isolates. These isolates were clustered as follows: *R. phaseoli* (9 isolates), *R. vallis* (1 isolate), *R. pisi* (2 isolates), and *R. etli* bv. *Phaseoli* (Fig. 5). GIE clustered all *Bradyrhizobium* species and 59% of *Vigna* isolates from Venezuela. These groups included the same isolates in the *nodD* phylogenetic tree (Table 3). However, the group of *B. japonicum* included 2 isolates previously classified as *Ensifer* (the 16S rRNA gene). The remaining isolates were β-rhizobia (GII) classified similarly to the *nodD* sequences analysis (Fig. 5). Furthermore, the TrV5 isolate was classified into the *Bradyrhizobium* group (GIE) without any reference strains. These results suggest that TrV5 is a novel lineage according to housekeeping and symbiotic gene sequences (Table 3).

### Symbiotic performance of Venezuelan isolates

The symbiotic performance of *Vigna* isolates is shown in Table 3. Forty-six isolates were inoculated onto *V. unguiculata* ‘Tuy’, and their effectiveness was recorded according to the interaction with the host. Root nodule numbers fluctuated between 9.0 and 100.0. FV3 showed the highest root nodule number (100.0±9.0) and MiR7 the second highest (95.0±4.8). These two isolates belonged to *Ensifer* and *Rhizobium*, respectively. LaV14 (*Burkholderia*) and ApV14 (*Bradyrhizobium*) showed the lowest nodulation. In the case of ARA, isolates ranged from being from ineffective to 100.0±2.9 μmol C_2_H_4_^–1^ h^–1^ g^–1^ per DW of nodules. The highest performance was displayed by AmR1 (from Amazonas) classified as *Rhizobium* and ineffective isolates were mainly related to *Burkholderia* or *Agrobacterium* (Table 3). The root nodules developed by *Rhizobium* isolates exhibited slightly higher ARA than those by other strains, such as *Agrobacterium* and some *Paraburkholderia* isolates. All isolates had a significantly larger biomass than the non-inoculated control (290.1±25.0 mg plant^–1^) by Dunnett’s tests. The exceptions were AFV2, AV19A, GR5, and MV1, which showed no significant differences. The highest biomass belonged to TrV2B with 2,283.8±100.0 mg plant^–1^. The isolate TrV2B was classified as *Bradyrhizobium* (Table 3). Additionally, no relationship was observed between nodule numbers and ARA by Tukey’s test (data not shown). However, a correlation was found between ARA plant^–1^ and biomass in comparisons of all samples (Table 3). Furthermore, the specific linear correlation (data not shown) showed a moderate correlation between biomass and ARA plant^–1^ because several strains did not have a relationship with these parameters, such as ApV1, AFV2, and LaV12 (Table 3).

## Discussion

### *Vigna*-rhizobia isolation, distribution, and physiological responses

The nodulation activity, geographical distribution, and diversity of rhizobia were mainly related to the history of legume cultivation and environmental factors, such as climate conditions and soil parameters, in the present study. The distribution of *Vigna*-rhizobia in Venezuela based on housekeeping genes revealed that α-rhizobia was dominant and widely distributed in Venezuelan soils. This result suggested that distribution was affected by the cultivation history of native or cultivated legumes such as *Vigna*. Cuadrado *et al.* (2009) reported that *V. unguiculata* was mainly nodulated by *Rhizobium*, *Bradyrhizobium*, and *Mesorhizobium* in Colombia. Our results are consistent with these findings, except for *Mesorhizobium* strains. Zhang *et al.* (2008) showed that in subtropical areas of China, the dominant group associated with *V. unguiculata* and *V. radiata* was *Bradyrhizobium*. Moreover, in Japan, Akatsu *et al.* (2014) isolated *Ensifer* and *Rhizobium* strains associated with *V. marina* and *V. radiata* from sandy soils.

As expected, Inceptisol (Aragua, Valley) showed a high rate of nodulation and Venezuelan rhizobia with a dominance of *Rhizobium* over *Bradyrhizobium*, and this may be attributed to the optimal agroecological conditions and continuous bean cultivation in these sites. Similarly, the *Rhizobium* group was dominant in Alfisol. However, the absence of nodulation in DC soils may be related to the lack of *Vigna* cultivation history, climate, and the combination of acidic pH and Al (Casanova, 2005). Furthermore, Venezuelan isolates classified as *Rhizobium* suggested a close relationship with *R. phaseoli* and *R. etli*. For example, AFV2 was classified as *R. phaseoli*, *Rhizobium* sp. TAC182^T^, or *R. leguminosarum bv. phaseoli*^T^. *Rhizobium* sp. TAC182^T^ was previously related to *R. phaseoli* (Santamaría *et al.*, 2017). These *Rhizobium* strains were correlated mainly with *Phaseolus* species and isolated from Latin American countries. Several isolates were related to *R. phaseoli* VIAD11^T^, which had been found in Meso-America (Mexico), through Central-America (Dominican Republic), to South-America (Argentina, Ecuador), and linked with Europe through Spain (Díaz–Alcántara *et al.*, 2014), and now in Venezuela with distribution in savannas, valleys and mountain areas. Accordingly, Marquina *et al.* (2011) described *Rhizobium* strains isolated from the Venezuelan root nodules of *Leucaena*, which survived at 2% NaCl.

Falcón showed a good rate of nodulation. Therefore, *Vigna*-rhizobia obtained from these locations were expected to be more stress-tolerant than other areas (Graham *et al.*, 1981; Graham, 1992). These Venezuelan isolates and reference strains were isolated under similar environmental conditions, such as temperatures of approximately 40°C, arid or semi-arid ecosystems with sandbanks, and Aridisol as the soil type. *E. mexicanus* ITTG-R7T (NR115768.1) was taken from Tuxtla in Mexico (Lloret *et al.*, 2007), while in Venezuelan, they were from Falcón. Venezuelan isolates (FV3, FV4, and FV6) and *E. mexicanus* reported by Lloret *et al.* (2007) strongly suggested that these *Ensifer* strains originated from American hosts, with a specific distribution under high temperatures and water stress conditions. Regarding *Vigna*, Akatsu *et al.* (2014) reported *Ensifer* strains with salinity tolerance under more than 3.5% NaCl that also grew well at 45°C. The salinity tolerance of isolates suggested a slight tendency according to the origin of isolates.

Isolates related to the genus *Bradyrhizobium* were dominant in Ultisol (Trujillo, Andes). These isolates showed pH tolerance, which suggests that Al and Mn toxicities as well as Ca^2+^ deficiencies are often associated with low soil pH (Graham *et al.*, 1981; Rodríguez and López, 2009; Guimarães *et al.*, 2012; Artigas *et al.*, 2019). In Venezuela, these types of soils are common in traditional cropping systems, such as in Trujillo where CaCO_3_ is added before and during *Vigna* cultivation (Casanova, 2005). Silva *et al.* (2014) reported that in the Brazilian rainforest, *Vigna* was mainly nodulated by *Bradyrhizobium*, which is consistent with the present results. However, the isolates classified as *Ensifer* and *Bradyrhizobium* showed more biogeographic specificity than *Rhizobium*.

On the other hand, some isolates were related to β-rhizobia and mainly associated with *V. radiata*, which is in contrast to previous findings (Zhang *et al.*, 2008; Risal *et al.*, 2012; Akatsu *et al.*, 2014; Bejarano *et al.*, 2014; Andrews and Andrews, 2017). *Paraburkholderia* isolates were obtained from nutrient-deficient soils, such as Guárico, Lara, and Falcón, as reported previously (Ferreira *et al.*, 2012; Radl *et al.*, 2014). Moreover, Guárico and Falcón have no previous cultivation history of *Vigna*. In tropical soils, a reduction in the rhizobial population size due to *Vigna* seeds occurred under successive stress nutrient conditions (Graham *et al.*, 1981). In Brazil, β-rhizobia has been described as a symbiont of *V. unguiculata* in semi-arid and Amazonian soils (Guimarães *et al.*, 2012; Radl *et al.*, 2014), but not in other countries, such as Africa and Asia (Sarr *et al.*, 2011). Venezuelan *Paraburkholderia* was identified by biogeographic relationship and soil fertility problems (Artigas *et al.*, 2019). In Latin-American countries, such as Mexico, Brazil, and Uruguay, *Burkholderia* has mainly been associated with *Mimosa* species (Bontemps *et al.*, 2016; Platero *et al.*, 2016). Some *Burkholderia* species have been reported as acid-tolerant and commonly distributed in low nutrient soils (Graham, 1992; De Oliveira–L *et al.*, 2015; Bontemps *et al.*, 2016; Platero *et al.*, 2016; Artigas *et al.*, 2019).

The optimal season for *V. unguiculata* and *V. radiata* cultivation significantly differs according to topographic and climatic regions in Venezuela. In the case of *Vigna* legumes, *V. unguiculata* is slightly more promiscuous than *V. radiata*, principally under field conditions. In the present study, no relationship was observed between strains isolated from both *Vigna* species and pH tolerance. However, pH conditions affected symbiosis, such as rhizobial growth, decreased nodule initiation, and impaired nodule function (Graham *et al.*, 1981; Casanova, 2005; Guimarães *et al.*, 2012). Additionally, the different pH tolerance of rhizobia influenced strain competitiveness (Sylvester–Bradley, 1983; Singh *et al.*, 1997). All factors were linked to N_2_ fixation in different ways and legume growth.

Legumes belonging to the *Phaseoleae* tribe, such as *V. unguiculata*, are nodulated by rhizobia from different genera across *α-* and *β-Proteobacteria* (Andrews and Andrews, 2017). Since *V. unguiculata* is indigenous to the South Africa Transvaal region, our results suggest that this legume is adapted for nodulation by different rhizobia species in Venezuelan soil (Velázquez *et al.*, 2010; Córdova–Sanchéz *et al.*, 2011; Parra, 2013; Chidebe *et al.*, 2018). In the present study, a new premise was suggested about these rhizobia, which could have a tropical or subtropical origin because the isolation was from *Phaseoleae* legumes. One possibility may be that the introduction, variation, and cultivation history of legumes in different regions forced the evolution of several root-nodulating bacteria, which may form symbioses under different conditions (Graham, 1992; Barcellos *et al.*, 2007; Córdova–Sanchéz *et al.*, 2011; Parra, 2013; Chidebe *et al.*, 2018). Furthermore, limited information is currently available on the diversity, distribution, and ecology of endemic Venezuelan legume symbionts. Despite their diversity and biotechnological potential, most of the bacterial strains isolated from Venezuelan soil remain uncharacterized.

### Symbiotic genes and horizontal symbiotic gene transfer

The distribution of *nod* genes depends on the relationship between or co-evolution of rhizobia and cultivars. Díaz–Alcántara *et al.* (2014) reported that Mesoamerican cultivars were associated with rhizobia that carried a *nod* allele originating in Mexico (North American), while Andean cultivars carried a *nod* allele from Ecuador (South America). These alleles could be identified according to the cultivar region of origin, *e.g.*, Colombian and Brazilian rhizobia possessed the same *nod* allele as rhizobia associated with beans in Ecuador. Therefore, Venezuelan isolates appear to carry a *nodD* allele that is related to the ecosystem and cultivar. Santamaría *et al.* (2017) reported that several unrelated strains, such as *Rhizobium* sp. TAC182^T^, have a pSyms plasmid that may converge or diverge toward environmental conditions and the nodulation of common hosts. This may explain the results with several strains between *Rhizobium* isolates and the reason why the strains carrying this allele that have been distributed into the Caribbean from seedlings-beans of South America (Díaz–Alcántara *et al.*, 2014).

Furthermore, previous studies reported that several symbiotic genes of *Mesorhizobium* were transferred from a symbiotic strain to different genera of rhizobia in the field (Sullivan and Ronson, 1998). Other studies demonstrated the horizontal transfer of symbiotic genes among an inoculant (*B. japonicum*) and indigenous Brazilian rhizobia, such as *B. elkanii* and *E. fredii*, in savanna soils (Barcellos *et al.*, 2007). This is consistent with the present results showing the existence of *nod* and *nif* genes between α-rhizobia genera. The relationship between *Agrobacterium* and *Rhizobium* may explain horizontal symbiotic gene transfer, in which the adaptation and co-evolution of legume-rhizobia interactions is an important factor (Paludosi and Ng, 1997; Velázquez *et al.*, 2010; Chidebe *et al.*, 2018). Furthermore, Quinto *et al.* (1985) reported three different regions in the symbiotic plasmid of *R. phaseoli*, which contained the complete coding sequence of the *nif* gene. The nucleotide sequences of the three *nif* genes were identical and functional copies (Quinto *et al.*, 1985). However, this finding implies that at least 2 of the reiterated genes were functionally expressed after transfer, which may have occurred between some of our isolates.

In the case of β-rhizobia, Venezuelan *Paraburkholderia* displayed nodulation, whereas ARA was not detected, which is consistent with previous findings in Brazil (Ferreira *et al.*, 2012; Silva *et al.*, 2014). Brazilian isolates related to *P. fungorum* were found in Amazonian soil (Brazil) using *P. vulgaris* (Ferreira *et al.*, 2012; Silva *et al.*, 2014), and explains the result obtained with LaV14 suggesting the presence of horizontal gene transfer between α- and β-rhizobia isolates in Venezuelan soils. *B. elkanii* was previously reported to be widely distributed in tropical soils (Barcellos *et al.*, 2007; Radl *et al.*, 2014). The appearance of nitrogen fixation genes from *B. elkanii* in *Paraburkholderia* suggests the existence of the horizontal gene transfer of symbiotic islands between *Bradyrhizobium* and *Paraburkholderia* (Artigas *et al.*, 2019). However, a comparative molecular analysis is needed to confirm this.

### Symbiotic function and plant growth promotion

The symbiotic performance of *Vigna* isolates and their effectiveness varied from ineffective to high plant growth promotion. Consequently, a relationship was not observed between nodulation and nitrogen fixation or biomass. Incompatibility between rhizobia and *Vigna* cultivars has been reported, for example, *V. radiata* ‘KPS1’ is incompatible with *B. elkanii* USDA61, resulting in ineffective nodulation, whereas this type of rhizobia is compatible with *V. mungo* (Nguyen *et al.*, 2018). These findings may explain the ineffectiveness observed in some *Bradyrhizobium* strains (*e.g.*, ApV14, AFV22, and TrV27) isolated from *V. unguiculata* at different sampling sites and inoculated on the same plant host. Moreover, the positive correlation between ARA (per plant) and biomass indicates that the number or mass of root nodules is an important factor for the utilization of energy by plants and the efficiency of strains (Cuadrado *et al.*, 2009).

Additionally, the results obtained for several α-rhizobia (*e.g.*, TrV2B) suggested that Venezuelan-rhizobia promoted plant growth by mechanisms other than nitrogen fixation, such as phytohormone production and phosphorus solubilization. Rodríguez and López (2009) inoculated *V. unguiculata* with plant growth-promoting bacteria (such as *Bacillus megaterium* and non-nodulating *Burkholderia*) in combination with a reduced amount of basal fertilizer (N, P, K,) in Ultisol (savanna, Guárico). The findings obtained showed that the biomass increased and a high capacity for rhizobia colonization was observed (Rodríguez and López, 2009), suggesting that native rhizobia contribute to the high biomass or healthy crop production. In the present study, two isolates had a slightly higher biomass and ARA (AmR1 and AFV15); one isolated without a *Vigna* cultivation history in Amazonas (rainforest), and the other with a legume cultivation history from Aragua (valley, with fertilization). Additionally, these isolates appeared to be genetically stable at symbiotic genes, and root nodule masses were lower than those in other samples, such as GV20, which reflected energy use by the plant. Accordingly, AFV15 showed several proprieties, such as fast growth and tolerance to high Al concentrations under acidic pH conditions. These results indicate the potential of these isolates as effective inoculants for *Vigna* plants under abiotic stress conditions.

In conclusion, the present results contribute to our understanding of the distribution and environmental conditions related to two *Vigna* species and their symbionts in Venezuela. In the present study, important issues that may affect rhizobial diversity and competitiveness were highlighted, such as crop production, soil conditions, and other factors. Additionally, the results obtained suggest that undisturbed soil covered with native vegetation may have a higher diversity of rhizobia. Furthermore, this study confirmed that an uncommon and understudied group of rhizobia (genus *Paraburkholderia*) was associated with *Vigna* plants in Venezuela. Some isolates showed different *nifH* and *nodD* alleles than expected from the housekeeping gene analysis, suggesting horizontal gene transfer. The highest symbiotic performance of AFV15 (*Rhizobium*) in combination with good physiological responses indicates its potential as a suitable novel inoculant for *Vigna* under abiotic stress conditions or possibly in combination with other K or P fertilizers. However, further field studies are needed to confirm the effectiveness of AFV15 as a *Vigna* inoculant.

## Supplementary Material

Supplementary Material 1

Supplementary Material 2

## Figures and Tables

**Table 1.** General characteristics of soil sampling and nodule numbers on *Vigna*

**Origin**	**State** (Sites)	**Cardinal location**	**Ecosystem**	**Soil type**	**pH**^a^	**Temp.** (°C)^b^	**Al** (ppm)**^c^**	**Vegetation**	**Total number** **nodules on*****Vigna***			**Total strains** **isolated from*****Vigna***	
									***unguiculata***	***radiata***		***unguiculata***	***radiata***
**Soils** **(Pot test)**	**Amazonas**	South	Jungle/rain-forest	Oxisol*	5.5~6.0	12~33	0.25	Cucumber, tomato, coriander, *Capsicum* sp. *Phaseolus* sp., Fabaceae*	32	22		20	12
	**Apure**	Southwest	Floodplain	Inceptisol	4.2~5.0	10~34	2.25	*Acacia* sp., *Caraipa* sp., *Mauritia* sp. *Tabebuia* sp., and Fabaceae*	22	0		17	0
	**Aragua**	North-central	Valley No Fertilizer	Inceptisol	7.1~7.5	18~31	0.00	*Sesamun* sp., *Vigna* sp., *Arachis* sp., *Canajus* sp.	52	20		15	3
	**Aragua**	North-central	Valley with Fertilizer	Inceptisol	7.5~8.0		0.00	*Asteracea* sp., *Phaseolus* sp., *G. max*, grasses	56	23		16	15
	**DC (Caracas)**	North-central	National Park in City	Alfisol	5.6~6.5	10~31	3.75	*Coffea* sp., sugarcane, tree forest, Bryophytes, Fabaceae*	44	0		22	0
	**Falcón**	Northwest	Arid-desert	Aridisol	8.0~10	22~40	1.13	*Prosopis* sp., *Opuntia* sp.	33	17		16	15
	**Guárico**	Central	Savanna	Vertisol	5.8~6.5	25~35	0.00	Cereals, Fabaceae*	21	14		20	6
	**Lara**	West-central	Dried savanna	Vertisol	6.0~6.3	10~34	0.38	*Coffea* sp., *Inga* sp., grasses, *Phaseolus* sp.	29	14		16	18
	**Mérida**	Southwest	Andes-Template	Ultisol	4.4~5.0	6±24	3.50	*Musa* sp., *Lactuca* sp., *Theobroma* sp., forestal species, *Phaseolus* sp.	38	24		17	12
	**Miranda**	Central coast	Mountain	Alfisol	5.6~6.0	10~33	0.00	Tree species of humid forest as *Pseudobombax*, Fabaceae*	37	14		19	10
	**Trujillo**	Northwest	Andes	Ultisol	3.6~4.5	10~24	20.38	*Theobroma* sp., *Inga edulis*	11	0		9	0
**Root nodules from the field in Venezuela**	**Aragua**	North-central coast	Valley No Fertilizer	Inceptisol	7.1~7.5	18~31	0.00	*Sorghum* sp., grasses, *Vigna*	45	0		5	0
	**Aragua**		Valley with Fertilizer	Inceptisol	7.5~8.0		0.00	*Asteracea* species, *Phaseolus*	35	0		1	0
	**Trujillo**	Northwest	Andes	Ultisol	3.6~4.5	10~24	20.38	*Coffea arabica, Zea* sp., Fabaceae*	20	0		3	0
								**Total**	**475**	**148**		**196**	**91**

^a^: These values were reported by Casanova (2005) and confirmed using a standard pH soil method (Jones, 2001; Casanova, 2005). ^b^: Temperature average reported by REDBC and INIA-Venezuela. ^c^: Al^3+^ method (Hsu, 1963; Jones, 2001). *: Amazon soil is classified as Entisol and Oxisol; however, the sampling site was Oxisol. Fabaceae*: It includes non-cultivated plants and unknown genera of trees, shrubs, and perennial or annual herbaceous plants.

**Table 2.** Number of isolates selected for different analyses and their distribution under different Venezuelan agro-conditions.

**State (Sites)**	**Soil type**	**For abiotic** **stress**	**For phylogenetic analysis**			**Bacterial classes**	
			***V. unguiculata***	***V. radiata***		***α-Proteobacteria***	***β-Proteobacteria***
**Amazonas**	Oxisol	10	3	2		5	0
**Apure**	Inceptisol	10	4	0		4	0
**Aragua**	Inceptisol	13	4	0		4	0
**Aragua**	Inceptisol	10	3	1		4	0
**DC (Caracas)**	Alfisol	10	2	0		2	0
**Falcón**	Aridisol	9	4	1		4	1
**Guárico**	Vertisol	12	4	2		4	2
**Lara**	Vertisol	15	2	2		3	1
**Mérida**	Ultisol	12	3	2		5	0
**Miranda**	Alfisol	10	2	1		3	0
**Trujillo**	Ultisol	9	4	0		4	0
	**Total**	120	35	11		42	4

**Table 3.** Summary of phylogenetic analyses and symbiotic performance of *Vigna-*rhizobia distributed in Venezuela

**Isolate name**	**Site: Soil type** (Ecosystem)^a^	**16S rRNA/*****atpD***	***nod*****D gene^b^**	***nif*****H***** gene***	**Biomass** (DW mg plant^–1^)	**Root nodule numbers^c^**	**Root nodule mass** (DW mg)	**ARA** (μmol plant^–1^)^d^	**ARA** (μmol Nod^–1^)^e^
**AmR1**	Amazonas: Oxisol (Am-r)	*Rhizobium* sp.	*R. leguminosarum*	*R. phaseoli*	1,709.0±29.0	30.0±2.9	3.6±0.7	0.4±0.2	100.0±2.9
**AmR3**		*Bradyrhizobium* sp.	*B. japonicum*	*Bradyrhizobium* sp.	1,258.3±25.0	59.0±1.4	128.3±18.1	7.5±1.4	57.5±1.4
**AmV1**		*R. pusense*	*B. elkanii*	*B. elkanii*	823.5±33.0	15.7±9.0	28.2±1.6	0.1±0.1	2.9±9.0
**AmV15**		*R. pusense*	*B. elkanii*	*B. elkanii*	481.2±39.0	31.7±7.0	40.8±1.7	0.2±0.03	5.4±7.0
**AmV5**		*R. pusense*	*Bradyrhizobium* sp.	*B. liaoningense*	419.2±17.0	13.5±1.0	112.5±11.0	0.0±0.0	0.0
**ApV1**	Apure: Inceptisol (F)	*R. tropici*	*R. leguminosarum*	*R. phaseoli*	1,779.0±102.0	37.5±0.1	98.8±3.8	0.5±0.1	5.0±0.1
**ApV14**		*Bradyrhizobium* sp.	*Bradyrhizobium* sp.	*B. yuanmingense*	648.7±6.5	11.0±0.5	32.7±10.7	0.0	0.0
**ApV4**		*B. embrapense*	*B. elkanii*	*B. embrapense*	610.2±70.0	30.7±1.5	43.4±1.6	0.3±0.1	7.5±1.5
**ApV8**		*Bradyrhizobium* sp.	*B. elkanii*	*B. elkanii*	521.5±100.0	40.0±1.0	37.0±3.0	0.3±0.1	8.9±1.0
**AV17^•^**	Aragua: Inceptisol (V-WF)	*Rhizobium* sp.	*R. leguminosarum*	*R. phaseoli*	1,309.2±8.5	43.5±0.5	89.5±0.5	1.8±0.4	19.6±0.5
**AV18**		*Agrobacterium* sp.	*R. leguminosarum*	*B. yuanmingense*	495.3±40.0	21.0±1.5	32.5±2.2	0.2±0.1	7.0±1.5
**AV19A^•^**		*Rhizobium* sp.	*R. leguminosarum*	*R. phaseoli*	289.5±26.0	29.5±0.5	91.1±10.0	0.9±0.1	9.7±3.0
**AV5**		*R. pusense*	*Bradyrhizobium* sp.	*B. yuanmingense*	1,242.0±220.0	35.3±0.7	51.7±6.0	0.6±0.1	12.5±0.7
**AFR13**	Aragua: Inceptisol (V-F)	*Bradyrhizobium* sp.	*B. elkanii*	*B. elkanii*	1,110.8±160.0	44.0±0.8	98.3±4.1	1.8±0.2	18.2±0.8
**AFV15^•^**		*Rhizobium* sp.	*R. leguminosarum*	*R. phaseoli*	1,783.5±20.0	87.7±3.0	26.1±4.8	2.0±0.1	79.3±0.5
**AFV2**		*Rhizobium* sp.	*R. leguminosarum*	*R. phaseoli*	234.7±12.0	16.0±0.1	12.7±2.3	0.5±0.2	37.6±1.0
**AFV22**		*Bradyrhizobium* sp.	*Bradyrhizobium* sp.	*B. yuanmingense*	364.0±57.0	34.5±0.3	70.0±8.9	0.1±0.01	0.7±0.3
**DCV11**	DC: Alfisol (N.P-C)	*R. vallis*	*R. leguminosarum*	*R. vallis*	1,237.8±300.0	52.7±9.0	71.0±20.0	0.01±0.03	7.8±5.0
**DCV2**		*Agrobacterium* sp.	*R. leguminosarum*	*B. liaoningense*	905.7±113.0	45.0±1.0	55.3±22.0	0.0	0.0
**FR1**	Falcon: Aridisol (A-D)	*Burkholderia* sp.	*Paraburkholderia* sp.	*P. phymatum	410.2±49.0	45.0±0.1	58.4±1.5	0.0	0.0
**FV16**		*R. pusense*	*B. elkanii*	*B. elkanii*	1,298.2±87.0	22.0±0.4	19.5±0.4	0.7±0.1	36.1±0.4
**FV3**		*E. mexicanus*	*Ensifer* sp.	*E. mexicanus*	1,497.8±46.0	100.0±9.0	117.0±15.2	1.4±0.5	11.8±9.0
**FV4**		*E. mexicanus*	*Ensifer* sp.	*B. japonicum*	709.0±97.0	22.5±1.3	12.0±3.6	0.5±0.1	44.7±1.3
**FV6**		*E. mexicanus*	*Ensifer* sp.	*B. japonicum*	432.1 ± 23.0	52.0±0.6	67.5±20.0	0.4±0.2	9.2±0.6
**GR3**	Guárico: Vertisol (S)	*Paraburkholderia* sp.	*Paraburkholderia* sp.	*P. phymatum	446.2±46.0	54.3±0.1	63.0±10.1	0.0	0.0
**GR5**		*Paraburkholderia* sp.	*Paraburkholderia* sp.	*P. phymatum	320.8±10.0	42.7±0.2	37.9±1.9	0.0	0.0
**GV1**		*Bradyrhizobium* sp.	*Bradyrhizobium* sp.	*Bradyrhizobium* sp.	1,081.7±48.0	44.0±6.9	57.1±22.8	0.9±0.03	19.3±6.9
**GV20**		*Rhizobium* sp.	*R. leguminosarum*	*R. phaseoli*	1,884.8±165.0	59.0±0.1	416.0±14.3	1.7±0.7	4.1±0.1
**GV5**		*Bradyrhizobium* sp.	*Bradyrhizobium* sp.	*Bradyrhizobium* sp.	512.8±46.0	47.5±0.1	60.1±16.9	0.1±0.02	2.4±0.1
**GV6**		*Rhizobium* sp.	*R. leguminosarum*	*R. phaseoli*	362.3±6.5	31.3±4.1	50.8±3.4	0.1±0.04	1.6±4.1
**LaR3**	Lara: Vertisol (D-S)	*B. embrapense*	*B. elkanii*	*B. embrapense*	972.8±15.0	70.5±5.5	69.3±9.6	0.2±0.1	2.9±2.0
**LaR7**		*Rhizobium* sp.	*B. elkanii*	*B. elkanii*	688.0±66.0	15.0±0.2	35.0±13.4	0.1±0.03	4.0±0.2
**LaV12**		*Rhizobium* sp.	*R. leguminosarum*	*R. mesoamericanun*	1,188.7±99.9	33.7±1.7	114.4±2.9	0.4±0.1	3.4±1.7
**LaV14**		*Paraburkholderia* sp.	*Paraburkholderia* sp.	*B. elkanii*	601.1±46.0	9.0±0.1	2.1±0.9	0.0	0.0
**MiR7**	Miranda: Alfisol (M)	*R. tropici*	*R. leguminosarum*	*R. tropici*	1,637.5±200.0	95.0±4.8	166.5±14.8	1.0±0.1	6.1±4.0
**MiV17**		*Rhizobium* sp.	*R. leguminosarum*	*Rhizobium* sp.	1,274.5±2.0	60.0±7.9	110.5±36.0	1.0±0.3	11.4±7.0
**MiV5**		*R. pisi*	*R. leguminosarum*	*R. pisi*	512.8±160.0	49.0±5.0	55.6±35.2	0.2±0.03	5.9±5.0
**MR7**	Mérida: Ultisol (A –T)	*Rhizobium* sp.	*R. leguminosarum*	*R. phaseoli*	938.7±120.0	42.0±2.8	69.3±8.3	0.4±0.05	6.3±2.8
**MV1**		*Agrobacterium* sp.	*R. leguminosarum*	*Rhizobium* sp.	249.1±16.0	41.0±8.0	66.0±5.4	0.5±0.05	7.1±2.0
**MR1**		*Bradyrhizobium* sp.	*B. elkanii*	*B. elkanii*	409.2±46.0	27.7±0.1	17.3±2.3	0.01±0.01	0.1±1.7
**MV3**		*Agrobacterium* sp.	*R. leguminosarum*	*B. liaoningense*	570.9±63.0	62.3±0.1	55.4±4.1	0.0	0.0
**MV7**		*B. embrapense*	*B. elkanii*	*B. embrapense*	777.8±97.0	37.5±0.4	90.8±0.7	1.0±0.2	11.0±0.4
**TrV26^•^**	Trujillo: Ultisol (A)	*Bradyrhizobium* sp.	*B. japonicum*	*Bradyrhizobium* sp.	1,583.3±68.0	31.0±9.0	9.0±0.9	0.4±0.2	50.4±9.0
**TrV27^•^**		*Bradyrhizobium* sp.	*Bradyrhizobium* sp.	*B. yuanmingense*	625.3±92.0	58.0±1.2	43.9±1.0	0.1±0.05	1.9±1.2
**TrV2B^•^**		*B. embrapense*	*B. elkanii*	*B. embrapense*	2,283.8±99.0	34.5±0.2	129.0±1.0	1.5±0.6	12.4±0.2
**TrV5**		*Bradyrhizobium* sp.	*Bradyrhizobium* sp.	*Bradyrhizobium* sp.	1,244.7±86.0	76.7±5.0	134.3±31.8	3.9±1.9	±5.0

•: These strains were isolated from field conditions. Sequences were compared using Blast in GenBank. *B.*: *Bradyrhizobium*. *P: *Paraburkholderia.* ^a^ V-F: valley with fertilizer; D-S: dried savanna; V-WF: valley without fertilizer; Am-r: Amazon – rainforest; A-D: aridic-like desert; A: Andes. A-T: Andes template. N.P.-C: National Park inside the city, F: floodplain. ^c^ Nodule numbers are per plant, 4‍ ‍weeks after inoculation (mean standard deviations; *n*=3). Control (non-inoculated) had no nodules. The plant test was performed with *V. unguiculata* ‘Tuy’. ^d^ Acetylene reduction assay (ARA). Values represent activity expressed as μmol C_2_H_4_^–1^ h^–1^ g^–1^ per plant and ^e^ ARA μmol C_2_H_4_^–1^ h^–1^ g^–1^ of nodules dry weight. Significant results based on *P*<0.05.
