# Supplementary material for: Phylogenetic Analysis of Symbiotic Bacteria Associated with Two Vigna Species under Different Agro-Ecological Conditions in Venezuela
Source: Microbes Environ. 2020 Jan 11;35(1):ME19120. doi: 10.1264/jsme2.ME19120 (PMC7104274; doi:10.1264/jsme2.ME19120)
Supplement: Supplementary file 2 — Supplementary Material 2 [file 35_19120_s2.pdf]

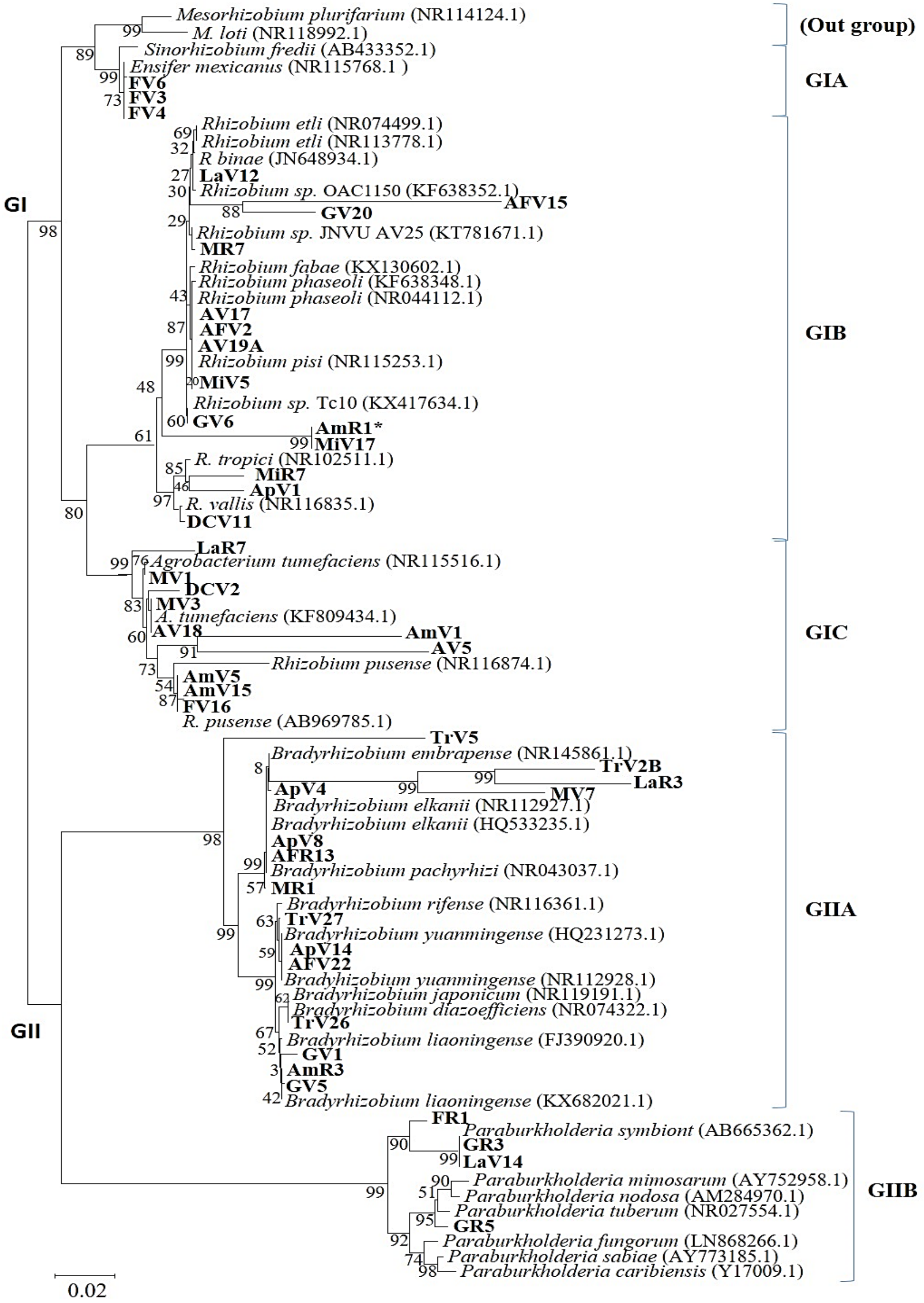

Fig. S1 Itemized phylogenetic tree based on 16S rRNA sequences of *Vigna*-rhizobia from different agro-ecosystem in Venezuela. Forty-six isolates and 38 references strains. Numbers at the nodes indicate the level of bootstrap support (%), based on a neighbor-joining analysis of 1,000 re-sampled datasets. The scale bar represents substitutions per nucleotide position.
